# 63. A case of enteropathic arthritis: or is it?

**DOI:** 10.1093/rap/rky034.026

**Published:** 2018-09-20

**Authors:** Magda Al-Zaza, Mohammed Abid Yusuf, Alexander Khakwani

**Affiliations:** Rheumatology, Colchester General Hospital NHS Trust, Colchester, UNITED KINGDOM

**Introduction:** A young lady presented with a progressive history of abdominal cramping and passing blood and mucus per rectum. She went on to develop peripheral arthritis and episcleritis, and saw multiple doctors in primary and secondary care who prescribed short courses of steroids which provided temporary relief. After an initial diagnosis of enteropathic arthritis, a new purpuric rash led to ANCA testing which confirmed granulomatosis with polyangiitis (GPA). This case reflects the need to keep an open mind regarding bowel symptoms suggestive of inflammatory bowel disease (IBD), as an alternative pathology may be the underlying problem.

**Case description:** A 21 year old white British lady initially presented to her GP in December 2017 with four months’ history of polyarthralgia involving her legs, shoulders, knees at night and pain and swelling of the small joints of the hands (proximal interphalangeal joint of middle and ring finger of left hand). This followed a two-year history of abdominal cramping pain with blood and mucus in the stool. Examination confirmed a positive MCP squeeze test of the left hand with difficulty making a fist. Left shoulder abduction was painful and limited. She was found to have mildly raised inflammatory markers CRP 25, ESR 27, was not anaemic and had normal renal and liver function. Rheumatoid factor was 23. She was prescribed co-codamol and referred to the rheumatology clinic, the appointment for which she did not attend. Two months later she presented to the emergency department with painful red eyes. An ophthalmologist noted bilateral episcleral nodules nasal and temporal with visual disturbance. She was diagnosed with bilateral episcleritis and treated with steroid eye drops with gradual weaning over a three week period. She was reviewed by gastroenterology two months afterwards for suspected IBD given her ongoing bowel complaint. She reported 2-3 stones of weight loss over the course of two months and a plan was made for a routine outpatient colonoscopy; a rheumatologist reviewed her at this point who advised sulfasalazine treatment for suspected enteropathic arthritis, only to be started after a colonoscopy. Interspersed between these clinical reviews, she saw different GPs and made two visits to the ED when she was prescribed 5-7 day courses of prednisolone 30mg daily, which helped her joint pain but not the abdominal pain. She presented to the ED a week after the rheumatology review with bilateral lower limb purpuric rash along with her ongoing abdominal cramps. Urine analysis showed protein and blood, and renal function was normal. The clinical suspicion was of Henoch Schönlein purpura (HSP). In view of her impending colonoscopy and suspicion of HSP, she was discharged without steroids with an ambulatory clinic review planned for two days later. She had ANA and ANCA checked for completeness due to the rash. In the ambulatory clinic, the rash showed improvement so she was discharged. Later that day, a call from the laboratory confirmed a positive cANCA with very high PR3 titre, confirming the diagnosis of GPA. The abdominal symptoms were therefore put down to mesenteric vasculitis and she was promptly started on treatment for ANCA associated vasculitis.

**Discussion:** A young patient presenting with abdominal cramping especially post-prandial, blood and mucus passage per rectum along with polyarthritis and inflammatory eye disease certainly would have IBD high on the differential diagnosis list. This seems to have led to the delay in diagnosing this lady correctly, and the turning point only came when she developed a purpuric rash. She likely managed to tolerate mesenteric ischaemia due to the vasculitis because of her young age and physiological reserve; CT mesenteric angiography did not show any evidence of necrosis, though she would have been at risk of this given the protracted nature of the symptoms. The value of keeping a wide differential diagnosis list when assessing a patient with a new purpuric rash is clear from this case. Although she was managed as having HSP, she had appropriate investigations simulataneously to rule out other causes.

**Key Learning Points:** A wide differential diagnosis should be kept in patients presenting with a constellation of symptoms and signs; no conclusion should be drawn before other options are effectively ruled out. The importance of continuity of care is underlined in this case - if the patient had seen the same GP on multiple visits, they may have realised there was more to this than met the eye. Not all diarrhoea with blood and mucus is IBD.

**Table:** Table 1: Blood test results

Blood Test	April 2018	Post treatment with methylprednisolone
CRP	223	<4
ESR	52	32
Hb	103	109
Platelets	600	525
eGFR	>90	>90
Creatinine	80	83
Liver function test	normal	normal
RF	23	
ANA	negative	
Myeloperoxidase (MPO)	0.8	
ANCA	cANCA Positive	
Proteinase 3 (PR3)	125.0	
Urine creatinine	39.4 (2.55-20)	
Urine total protein	>6.00 g/l	
Urine protein/creatinine ratio	>152.3 mg/mmol (0-15)	
Urine albumin	>4400.0 mg/l	
Urine albumin/creatinine ratio	>111.7 mg/mmol (0-2.5)	

**Disclosure: M. Al-Zaza:** None. **M. Yusuf:** None. **A. Khakwani:** None.

